# Response of Lodging Resistance and Grain Yield to EDAH and Different Fertilization Combinations in Maize (*Zea mays* L.)

**DOI:** 10.3390/plants14233707

**Published:** 2025-12-04

**Authors:** Yuru Wang, Yifei Wang, Chenyang Jiang, Yuwen Liang, Genji You, Jian Guo, Dalei Lu, Guanghao Li

**Affiliations:** 1 Jiangsu Key Laboratory of Crop Genetics and Physiology, Jiangsu Co-Innovation Center for Modern Production Technology of Grain Crops, Yangzhou University, Yangzhou 225009, China; 17851971675@163.com (Y.W.); yzuwyfzs@163.com (Y.W.); 13646104102@163.com (C.J.); lyw17866710733@163.com (Y.L.); a2728152904@163.com (G.Y.); guojian90816@126.com (J.G.); dllu@yzu.edu.cn (D.L.); 2 Joint International Research Laboratory of Agriculture and Agri-Product Safety, The Ministry of Education of China, Yangzhou University, Yangzhou 225009, China

**Keywords:** maize, grain yield, EDAH, stem mechanical properties, fertilization methods, plant morphology

## Abstract

Stalk lodging is one of the major constraints limiting global maize yield. Chemical regulation and fertilization are essential agronomic practices that play critical roles in improving maize yield and lodging resistance. This study aimed to investigate the effects of different fertilization methods on maize plant morphology, stem mechanical properties and chemical composition, and yield under spraying chemical regulator (EDAH, consist of 27% ethephon and 3% DA-6). The experiment was conducted from 2023 to 2025, using Jiangyu668 (JY668) and Jiangyu877 (JY877) with different plant heights. Three fertilization methods (no fertilization, N0; conventional fertilization, N15; and slow-release fertilization, SN15) were set up. Chemical regulation and fertilization methods had significant effects on plant morphology, stem mechanical properties and chemical composition, lodging rate, and grain yield. The combination of spraying EDAH and slow-release fertilization optimized ear position coefficient and gravity center, decreased stem–leaf angle, and increased leaf orientation value, which was beneficial for improving leaf photosynthetic capacity. EDAH and slow-release fertilization also increased the stem internode diameter and aerial root layers; enhanced bending resistance and puncture strength; and increased cellulose, hemicellulose, and lignin contents and the lodging resistance index. These changes synergistically increased grain number and weight, ultimately increased maize yield, and decreased the lodging rate. CSN15 had highest yield and lowest lodging rate in different years and varieties. SN15 increased yield by 10.58% compared with N15, and CSN15 increased yield by 10.53% compared with CN15. JY877, as a medium- to high-stem maize variety, had better performance in plant morphology and yield than JY668 (dwarf maize variety) under EDAH and slow-release fertilization. These findings demonstrate that the strategy of combining chemical regulation and slow-release fertilization represents an optimal management approach for enhancing grain yield by optimizing plant morphology and improving stem mechanical properties and stem chemical composition in maize production. This strategy can increase agricultural productivity by enhancing yield and lodging resistance and provide significant environmental benefits and a scientific basis for agronomic practice recommendations.

## 1. Introduction

The frequent occurrence of extreme weather events and lodging in crops has posed significant challenges to crop stability and high-yield production, thereby threatening global food security [1]. As the world’s second largest maize producer, China has contributed 23% of the global maize output and has played a critical role in stabilizing global maize supply [2]. Increasing planting density is a vital approach to improving maize yields. However, high-density planting often results in competition for water, nutrients, and light, which leads to plant lodging [3,4]. Maize lodging significantly reduced grain yield, caused economic losses, deteriorated grain quality, complicated crop management, hindered mechanized harvesting, and increased harvesting costs [5,6,7]. Currently, three main strategies exist for mitigating maize lodging domestically and internationally. One key approach focuses on breeding lodging-resistant varieties with well-developed root systems, strong stems, and shorter plant heights [8,9]. The second strategy focuses on spraying chemical regulators to optimize plant morphology, enhance stem mechanical strength, improve the anatomical and biochemical properties of stems, and increase root anchorage capacity, thereby strengthening lodging resistance [10,11]. The third approach emphasizes adopting efficient fertilizer management strategies [5], improving yield and N use efficiency while simultaneously reducing the risk of stem lodging [12,13].

Chemical regulation technology modulates endogenous hormone levels in plants, precisely influencing growth and development to achieve stable yields, improved quality, and enhanced stress resistance [4,14,15], and is widely applied in agricultural production. In maize cultivation, chemical regulation enhanced lodging resistance by modifying morphological, physiological, and biochemical characteristics, thereby improving light use efficiency [16] and strengthening root support capacity [6,8,14,17]. A commonly used chemical regulator including 27% ethephon and 3% DA-6 (EDAH) is widely applied to enhance maize yield and lodging resistance [18,19]. EDAH significantly reduced maize plant height and the length of basal internodes while increasing stem diameter and the dry matter weight of the basal internodes [6,20,21]. Mechanical properties of maize basal internodes, such as bending resistance and puncture strength, were also enhanced, accompanied by significant increases in diameter and dry matter weight from the third to fifth internodes [11,22,23], and the stem cross-sectional area, cortex thickness, and both the number and area of vascular bundles were markedly increased [18,19]. These improvements directly strengthened stem mechanical resistance, contributing to enhanced lodging resistance [24]. EDAH delayed ear leaf senescence, increased the chlorophyll content [19,25], and optimized canopy light distribution. Characterized by a reduction in leaf area of the middle and lower canopy and an expansion in leaf area of the upper canopy, this distribution pattern established a foundation for improved photosynthetic efficiency and increased grain yield [22,26,27]. EDAH significantly enhanced the content of structural carbohydrates, including cellulose, hemicellulose, and lignin, in maize stems [18,19,28]. These structural components enhanced stem mechanical integrity, thereby improving its lodging resistance [29]. Meanwhile, the accumulation of non-structural carbohydrates (such as glucose and starch) provided nutritional reserves for maize plants [6,30], thereby promoting stress recovery, delayed senescence, and increased grain yield [1]. Under high planting density, root spatial expansion was restricted, leading to reduced anchorage capacity [10,31]. EDAH significantly enhanced root quantity, diameter, angle, volume, and dry weight [32,33]. This optimization strengthened root anchorage capacity and promoted functional coordination between roots and shoots, thereby providing critical support for stable yield, increasing productivity, and enhancing lodging resistance [4,14,18].

Efficient fertilizer management also played a critical role in maintaining or improving crop yield and quality, enhancing fertilizer use efficiency, and strengthening plant resistance [34]. Slow-release fertilizer application was an effective nutrient management strategy that not only enhanced nutrient use efficiency and maize yield but also significantly reduced labor costs [35]. In addition, slow-release fertilizers released N more gradually during the early growth stages of crops, which effectively delayed excessive elongation of basal internodes in maize, thereby reducing the risk of lodging [36]. Previous research showed that the application of slow-release fertilizers significantly improved soil properties by enhancing aggregate stability, reducing bulk density, promoting microbial activity, and increasing soil nutrient content [37,38,39]. Under medium planting density, one-time application of slow-release fertilizer promoted dry matter accumulation during the grain-filling stage and facilitated its efficient translocation to maize grains. Slow-release fertilizer also delayed leaf senescence and improved the source–sink ratio and harvest index, thereby significantly increasing grain yield [40,41]. By optimizing the synchronization of N supply and demand, slow-release fertilizer significantly reduced maize plant and ear height [13]; optimized the morphological characteristics of the second, third, and fourth basal internode; and significantly enhanced stem mechanical properties [42,43].

Although numerous studies have reported the regulatory effects of chemical growth regulators on lodging resistance in maize, as well as the impacts of different fertilization methods on maize growth and development, the combined effects of fertilization regimes and EDAH spraying on maize yield and lodging-related traits remain unclear. Therefore, this study aimed to systematically evaluate the effects of different fertilization methods (slow-release fertilizer and conventional fertilizer) combined with EDAH application on grain yield, plant morphology, stem mechanical properties, and stem chemical composition in two maize hybrids (JY668 and JY877). Furthermore, the study sought to elucidate the mechanisms underlying improvements in lodging resistance and yield potential, thereby providing theoretical and technical support for the cultivation of high-yielding, efficient, and lodging-resistant maize.

## 2. Results

### 2.1. Grain Yield and Yield Components

**Figure 1:**
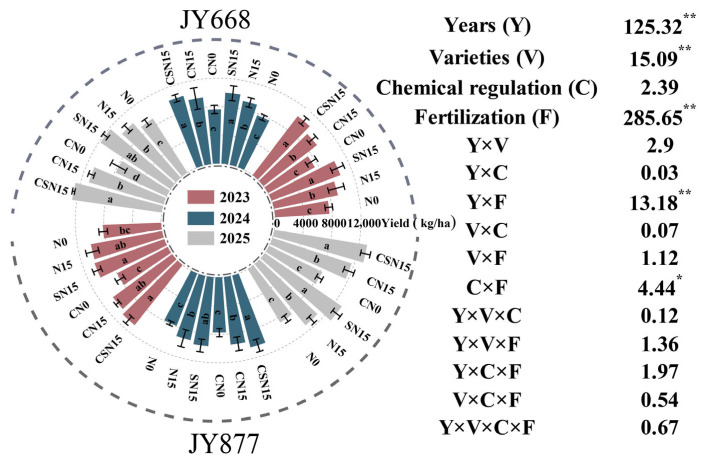
Response of maize yield to EDAH and different fertilization combinations from 2023 to 2025. N0: no fertilization; N15: conventional fertilization; SN15: one-time application of slow-release fertilizer; CN0: spraying EDAH under no fertilization; CN15: spraying EDAH under conventional fertilization; CSN15: spraying EDAH under one-time application of slow-release fertilizer. Multiple comparisons among treatment means were performed using Tukey’s HSD post hoc test. Error bars represent the mean ± SD. Different letters indicate significant differences among treatments. * and ** denote significance at *p* < 0.05 and *p* < 0.01 probability levels, respectively.

**Figure 2:**
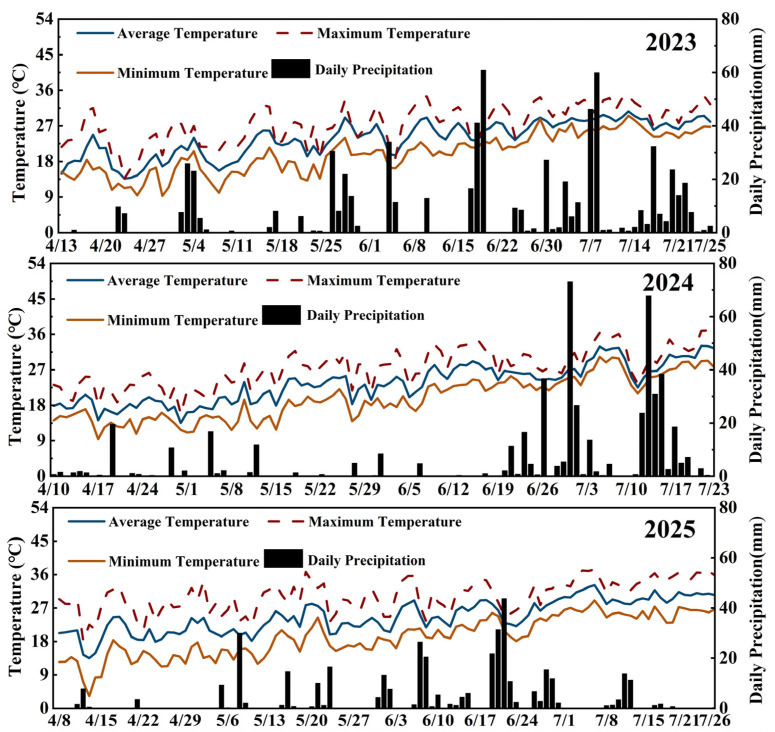
Temperature and precipitation during maize growing seasons from 2023 to 2025.

Varieties, fertilization methods, and years significantly influenced maize yield (Figure 1). Fertilization methods significantly increased maize yield, with SN15 having the highest yield. The average yields of SN15 were 12.3% (JY668) and 8.9% (JY877) higher than N15. The yields of CSN15 were 11.9% (JY668) and 9.2% (JY877) higher than CN15. The average yield of CSN15 was 3.6% higher than SN15, and that of CN15 was 3.7% higher than N15, but there was no significant difference. The highest yield was observed under the CSN15 treatment in JY877, mainly due to the synergistic effect of EDAH and fertilization methods, which increased the number and weight of grains per ear (Table 1). JY877 had a higher average yield than JY668. The highest yield was recorded in 2025, whereas the lowest was recorded in 2024, which may be attributed to excessive precipitation (401.35 mm) during the tasseling to maturity period (June 18 to July 23) in 2024 (Figure 2).

### 2.2. Plant and Ear Height and Gravity Center Height

**Figure 3:**
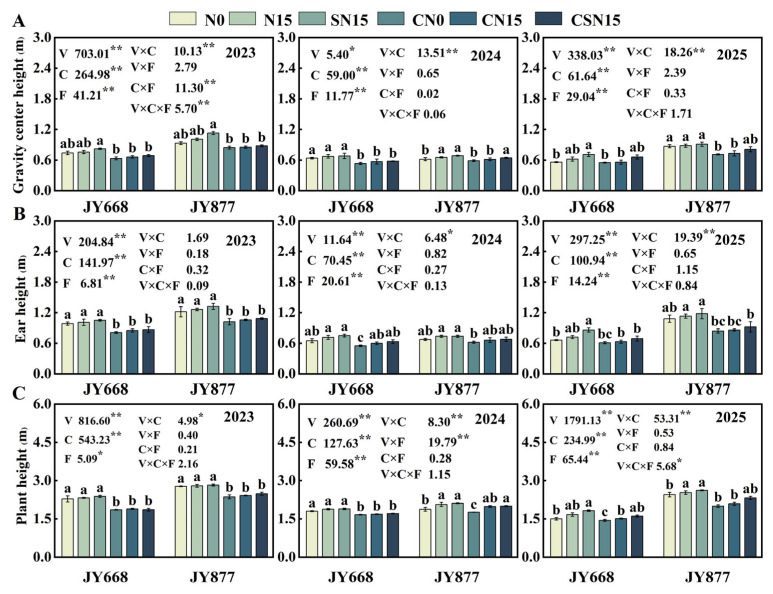
Response of maize plant and ear height and gravity center height to EDAH and different fertilization combinations from 2023 to 2025. N0: no fertilization; N15: conventional fertilization; SN15: one-time application of slow-release fertilizer; CN0: spraying EDAH under no fertilization; CN15: spraying EDAH under conventional fertilization; CSN15: spraying EDAH under one-time application of slow-release fertilizer. Multiple comparisons among treatment means were performed using Tukey’s HSD post hoc test. Error bars represent the mean ± SD. Lowercase letters indicate significant differences (*p* < 0.05) among different fertilization treatments within the same variety under chemical regulation. * and ** denote significance at *p* < 0.05 and *p* < 0.01 probability levels, respectively. (**A**) Gravity center height; (**B**) ear height; (**C**) plant height.

Spraying EDAH significantly decreased plant and ear height and gravity center height (*p* < 0.01). Average plant height, ear height, and gravity center height decreased by 13.2%, 15.6%, and 12.3% for JY668 and by 11.9%, 17.2%, and 13.2% for JY877. Plant height, ear height, and gravity center height in CSN15 decreased by 12.2%, 17.4%, and 13.9% compared to SN15, and those of CN15 decreased by 12.7%, 16.4%, and 12.9% compared to N15 (Figure 3). Fertilization methods had significant effects on plant height, ear height, and gravity center height (*p* < 0.01). Specifically, SN15 increased plant height, ear height, and gravity center height by 2.9%, 7.1%, and 7.9% in JY668 and by 3.5%, 3.7%, and 6.8% in JY877 compared to N15. There were significant differences in plant height, ear height, and gravity center height between the two varieties, with JY877 being higher than JY668. Overall, plant height, ear height, and gravity center height were the highest in 2023 and the lowest in 2024. This variation may be associated with environmental changes caused by increased precipitation and elevated temperatures during the critical growth period in 2024 (Figure 2).

### 2.3. Stem–Leaf Angle and Leaf Orientation Value

**Figure 4:**
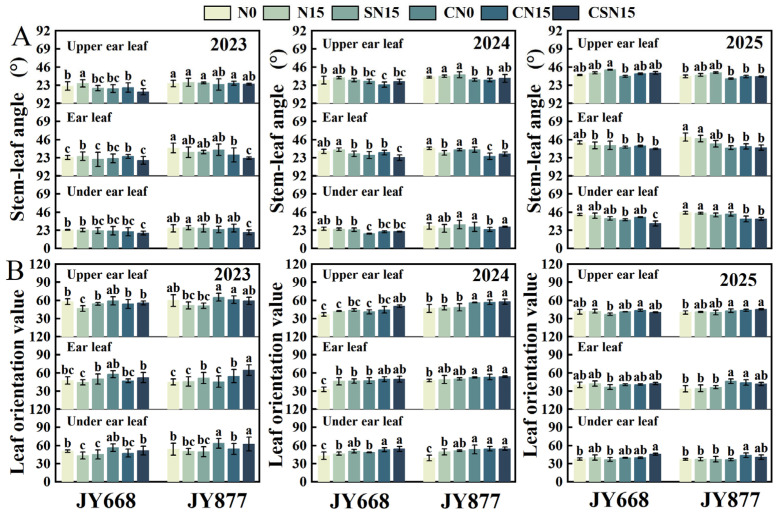
Response of maize stem–leaf angle and leaf orientation value to EDAH and different fertilization combinations from 2023 to 2025. N0: no fertilization; N15: conventional fertilization; SN15: one-time application of slow-release fertilizer; CN0: spraying EDAH under no fertilization; CN15: spraying EDAH under conventional fertilization; CSN15: spraying EDAH under one-time application of slow-release fertilizer. Multiple comparisons among treatment means were performed using Tukey’s HSD post hoc test. Error bars represent the mean ± SD. (**A**) stem–leaf angle; (**B**) leaf orientation value. Lowercase letters indicate significance at *p* < 0.05.

Spraying EDAH decreased the stem–leaf angle of the upper ear leaf (14.4%), ear leaf (9.5%), and under ear leaf (9.8%) in JY668, and in JY877, the decreases were 9.8%, 16.2%, and 8.3%, respectively (Figure 4A). Compared to N15, SN15 decreased the upper-ear and ear stem–leaf angles by 11.9% and 12.9% in JY668, respectively. Fertilization methods had no significant effect on the stem–leaf angle of JY877. Compared to CN15, CSN15 decreased the upper-ear and ear stem–leaf angles by 11.2% and 16.3% in JY668 and decreased the ear and under-ear stem–leaf angles by 2.6% and 2.4% in JY877, respectively. Compared to SN15, CSN15 decreased the stem–leaf angle of the upper-ear leaf (12.7%), ear leaf (14.8%), and under-ear leaf (12.3%). Compared to N15, CN15 decreased the stem–leaf angle of the upper-ear leaf (14.2%), ear leaf (11.1%), and under-ear leaf (6.1%). The stem–leaf angle of JY877 was generally greater than that of JY668. For both varieties, the overall pattern of the stem–leaf angle was ear leaf > upper leaf > lower leaf. The largest stem–leaf angle was observed in 2025, whereas the smallest occurred in 2023.

Spraying EDAH increased the leaf orientation value by 8.7% (upper-ear leaf), 10.4% (ear leaf), and 10.2% (under-ear leaf) in JY668 and by 14.4%, 16.2%, and 14.6% in JY877 (Figure 4B). Compared to CN15, CSN15 increased the leaf orientation values by 3.0% (upper-ear leaf), 5.2% (ear leaf), and 4.1% (under-ear leaf). Compared to SN15, CSN15 increased the leaf orientation values of the upper-ear leaf (14.5%), ear leaf (14.2%), and under-ear leaf (16.1%). Compared to N15, CN15 increased the leaf orientation values of the upper-ear leaf (13.0%), ear leaf (10.3%), and under-ear leaf (8.8%). Fertilization methods had no significant effect on the leaf orientation value. The leaf orientation value of JY877 was greater than that of JY668, with the highest value observed in 2023 and the lowest in 2025.

### 2.4. Internode Traits and Aerial Root Layers

**Figure 5:**
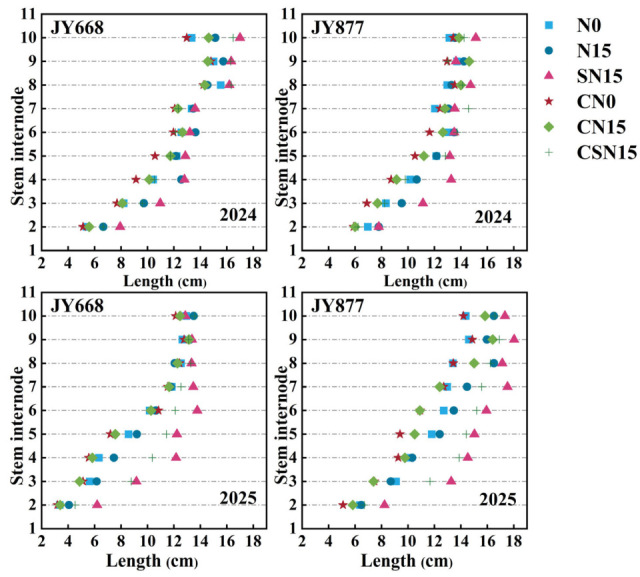
Response of maize stem internode length to EDAH and different fertilization combinations in 2024 and 2025. N0: no fertilization; N15: conventional fertilization; SN15: one-time application of slow-release fertilizer; CN0: spraying EDAH under no fertilization; CN15: spraying EDAH under conventional fertilization; CSN15: spraying EDAH under one-time application of slow-release fertilizer.

**Figure 6:**
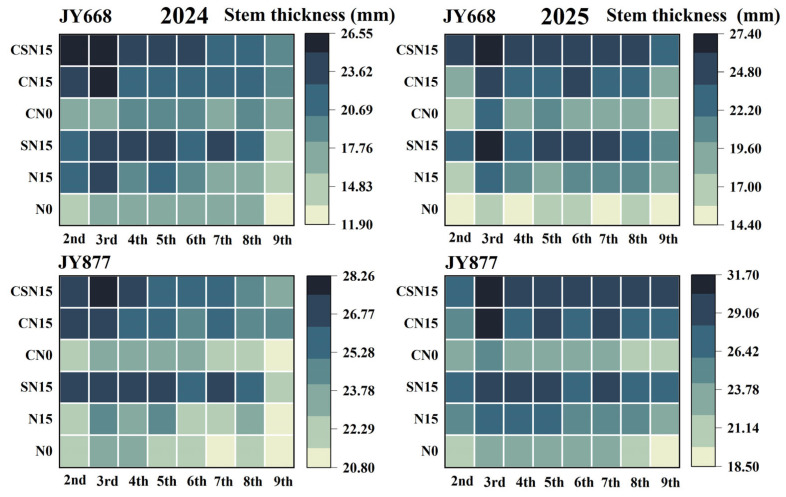
Response of maize internode diameter to EDAH and different fertilization combinations in 2024 and 2025. N0: no fertilization; N15: conventional fertilization; SN15: one-time application of slow-release fertilizer; CN0: spraying EDAH under no fertilization; CN15: spraying EDAH under conventional fertilization; CSN15: spraying EDAH under one-time application of slow-release fertilizer.

Spraying EDAH significantly decreased the maize internode length (Figure 5), mainly affecting the 2nd–5th internodes. Specifically, the lengths of the 2nd–5th internodes decreased by 15.2%, 11.4%, 16.5%, and 10.4% in JY668 and by 14.1%, 12.2%, 6.8%, and 12.4% in JY877, respectively. Compared with N15, SN15 mainly increased the length of the 2nd–5th internodes, with increases of 20.4%, 30.4%, 25.1%, and 17.7%, respectively. Spraying EDAH increased the maize internode diameter (Figure 6); compared with SN15, CSN15 increased the diameter of the 2nd–4th internodes by 6.7%, 6.1%, and 2.6%, respectively, and compared with N15, CN15 increased the diameter of the 2nd–5th internodes by 9.8%, 11.0%, 5.6%, and 7.4%, respectively. These results indicate that spraying EDAH primarily enhanced the diameter of the 2nd–4th internodes. Fertilization methods significantly affected the internode diameter. Compared with N15, SN15 increased the diameter of the 2nd–7th internodes by 12.4%, 7.1%, 12.0%, 9.0%, 8.6%, and 10.6%, respectively. Spraying EDAH had significant effects on the dry weight, length, diameter, and dry weight per unit length of the third internode (Table 2), with the dry weight per unit length increasing by 53.5% (JY668) and 24.3% (JY877). Compared with CN15, CSN15 increased the dry weight per unit length by 18.9%. Overall, the dry weight per unit length of JY877 was higher than that of JY668, and it was higher in 2025 than 2024.

**Figure 7:**
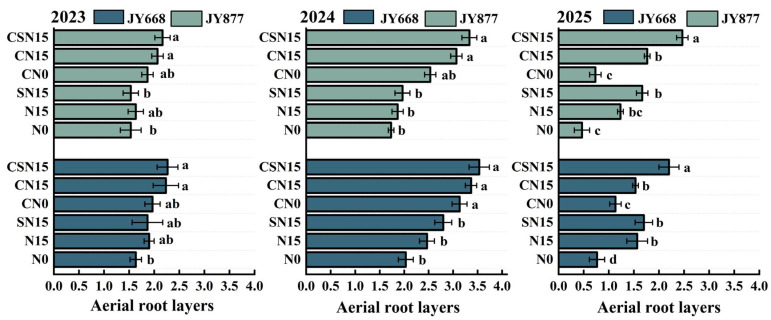
Response of maize root aerial layers to EDAH and different fertilization combinations from 2023 to 2025. N0: no fertilization; N15: conventional fertilization; SN15: one-time application of slow-release fertilizer; CN0: spraying EDAH under no fertilization; CN15: spraying EDAH under conventional fertilization; CSN15: spraying EDAH under one-time application of slow-release fertilizer. Multiple comparisons among treatment means were performed using Tukey’s HSD post hoc test. Error bars represent the mean ± SD. Lowercase letters indicate significant differences (*p* < 0.05) among different fertilization treatments within the same variety under chemical regulation.

Spraying EDAH increased the average number of aerial root layers by 0.51 in JY668 and by 0.71 in JY877 (Figure 7). CSN15 increased the number of aerial root layers by 0.74 compared to SN15, and CN15 increased the number by 0.56 compared to N15. JY668 exhibited 0.25 more aerial root layers than JY877 on the two-year average. Overall, the number of aerial root layers was the highest in 2024 and the lowest in 2023, which may be related to differences in cumulative temperature and precipitation from the jointing stage to the flowering stage. During this period in 2023, the cumulative temperature and total precipitation were 1323.7 °C and 236.1 mm (April 13 to June 13), whereas in 2024, they were 1264.3 °C and 96.9 mm (April 10 to June 10). The higher temperature and precipitation during this growth may have inhibited aerial root formation in 2023 (Figure 2).

### 2.5. Stem Mechanical Properties

**Figure 8:**
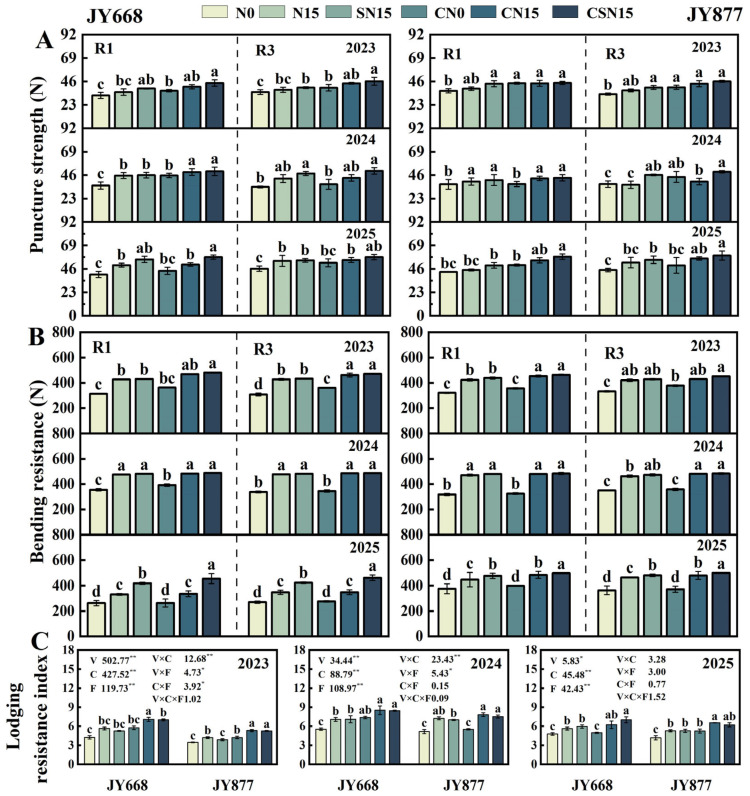
Response of maize stem mechanical strength to EDAH and different fertilization combinations from 2023 to 2025. Values are presented as mean ± SD (n = 3). N0: no fertilization; N15: conventional fertilization; SN15: one-time application of slow-release fertilizer; CN0: spraying EDAH under no fertilization; CN15: spraying EDAH under conventional fertilization; CSN15: spraying EDAH under one-time application of slow-release fertilizer; R1: silking stage; R3: milk stage. Multiple comparisons among treatment means were performed using Tukey’s HSD post hoc test. Lowercase letters indicated significant differences (*p* < 0.05) among different fertilization treatments within the same variety under chemical regulation. * and ** denote significance at *p* < 0.05 and *p* < 0.01 probability levels, respectively. (**A**) Puncture strength; (**B**) bending resistance; (**C**) lodging resistance index.

EDAH and fertilization methods had significant effects on bending resistance and puncture strength (Figure 8). Spraying EDAH increased bending resistance and puncture strength by 6.1% and 9.3% in JY668 and by 4.6% and 11.7% in JY877, respectively. Compared with N15, SN15 increased bending resistance and puncture strength by 7.4% and 6.5% in JY668 and by 3.3% and 10.4% in JY877, respectively. Compared with CN15, CSN15 increased bending resistance and puncture strength by 6.2% and 7.6%, respectively; compared with SN15, CSN15 increased these traits by 6.5% and 8.4%, respectively. Compared with N15, CN15 increased bending resistance and puncture strength by 3.9% and 6.5%, respectively. JY877 exhibited higher bending resistance than JY668, while puncture strength showed no significant difference between varieties. Under CSN15 treatment, both JY668 and JY877 reached the highest levels of bending resistance and puncture strength. Bending resistance was the highest in 2024 and the lowest in 2025, whereas puncture strength was the highest in 2025 and the lowest in 2023.

EDAH, fertilization methods, and varieties had significant effects on the lodging resistance index (*p* < 0.01). Spraying EDAH increased the lodging resistance index by 21.7% in JY668 and 17.5% in JY877. Compared with SN15, CSN15 increased the lodging resistance index by 20.3%, while compared with N15, CN15 increased it by 18.3%. Compared with N15, SN15 increased puncture strength by 18.9% in JY668 and by 17.1% in JY877. JY668 exhibited a higher lodging resistance index than JY877. Across years, the lodging resistance index was the highest in 2024 and the lowest in 2023.

### 2.6. Stem Chemical Composition

**Figure 9:**
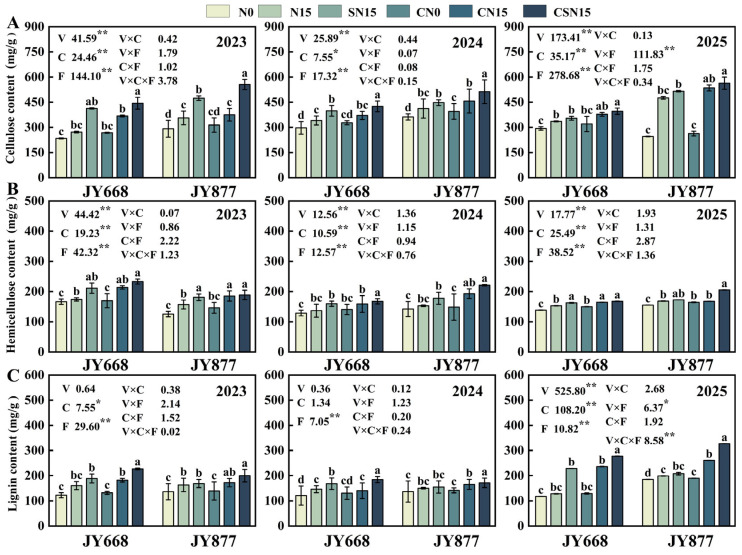
Response of stem chemical composition to EDAH and different fertilization combinations from 2023 to 2025 in spring maize. N0: no fertilization; N15: conventional fertilization; SN15: one-time application of slow-release fertilizer; CN0: spraying EDAH under no fertilization; CN15: spraying EDAH under conventional fertilization; CSN15: spraying EDAH under one-time application of slow-release fertilizer. Multiple comparisons among treatment means were performed using Tukey’s HSD post hoc test. Error bars represent the mean ± SD. Lowercase letters indicate significant differences (*p* < 0.05) among different fertilization treatments within the same variety under chemical regulation. * and ** denote significance at *p* < 0.05 and *p* < 0.01 probability levels, respectively. (**A**) Cellulose content; (**B**) hemicellulose content; (**C**) lignin content.

EDAH and fertilization methods had significant effects on cellulose, hemicellulose and lignin contents in the third internode of maize (Figure 9). Spraying EDAH increased cellulose, hemicellulose, and lignin contents by 12.2%, 9.4%, and 8.7% in JY668 and by 10.9%, 13.1%, and 7.9% in JY877 between two-year averages. Compared to N15, SN15 increased cellulose, hemicellulose, and lignin contents by 22.9%, 15.1%, and 3.5% in JY668 and by 15.4%, 11.0%, and 3.8% in JY877. Compared to CN15, CSN15 increased cellulose, hemicellulose, and lignin contents by 16.6%, 9.3%, and 19.9%, respectively. Compared to N15, CN15 increased cellulose, hemicellulose, and lignin contents by 13.3%, 14.9%, and 22.1%. Compared to SN15, CSN15 increased cellulose, hemicellulose, and lignin contents by 11.3%, 11.2%, and 24.2%, respectively. CSN15 treatment had the highest cellulose, hemicellulose, and lignin contents both in JY668 and JY877. Cellulose and hemicellulose contents differed significantly between the two varieties (*p* < 0.01), with those in JY877 being higher than in JY668. Lignin showed no significant difference between 2023 and 2024 (*p* > 0.05). The cellulose content was the highest in 2024 and the lowest in 2023. The hemicellulose content was the highest in 2023 and the lowest in 2024. The lignin content was the highest in 2025 and the lowest in 2024.

### 2.7. Lodging Rate

**Figure 10:**
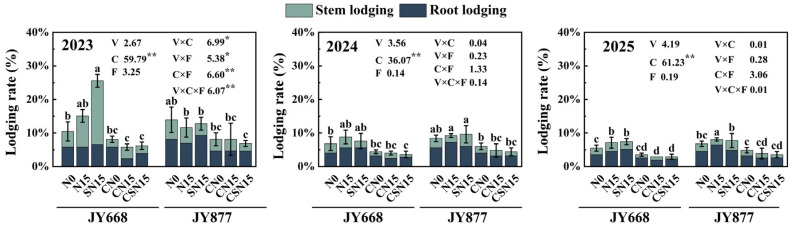
Response of maize lodging rate to EDAH and different fertilization combinations from 2023 to 2025. N0: no fertilization; N15: conventional fertilization; SN15: one-time application of slow-release fertilizer; CN0: spraying EDAH under no fertilization; CN15: spraying EDAH under conventional fertilization; CSN15: spraying EDAH under one-time application of slow-release fertilizer. Multiple comparisons among treatment means were performed using Tukey’s HSD post hoc test. Error bars represent the mean ± SD. Lowercase letters indicate the analysis of differences in total lodging rate (*p* < 0.05) among different fertilization treatments within the same variety under chemical regulation. * and ** denote significance at *p* < 0.05 and *p* < 0.01 probability levels, respectively.

EDAH had a significant effect on the lodging rate (*p* < 0.01). Spraying EDAH reduced the lodging rate of JY668 and JY877 by 56.0% and 42.6%, respectively (Figure 10). Compared with CN15, CSN15 reduced the lodging rate of JY877 by 11.7%, while no significant effect was observed in JY668. Compared with N15, CN15 reduced the lodging rate by 50.7%, and compared with SN15, CSN15 reduced the lodging rate by 61.1%. The lowest lodging rates for both JY668 and JY877 were observed under the CSN15 treatment. In 2024 and 2025, JY877 exhibited higher lodging rates than JY668. In 2023, JY668 showed higher lodging rates under the SN15 and N15 treatments, which may have been due to the occurrence of stem rot at maturity. Overall, lodging incidence was higher in 2023 than in 2024 and 2025, which may be related to higher precipitation during the growing season (Figure 2).

### 2.8. Correlation Analysis and Path Analysis

**Figure 11:**
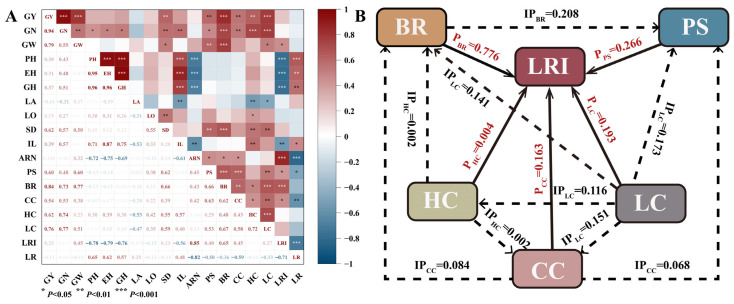
Correlation analysis and path analysis between maize yield and lodging resistance traits. GY, grain yield. GN, grain number per ear. GW, 1000-grain weight. PH, plant height. EH, ear height. GH, gravity center height. LA, stem–leaf angle. LO, leaf orientation value. SD, diameter of third internode stem. IL, length of third internode stem. ARN, number of aerial root layers. PS, puncture strength. BR, bending resistance. CC, cellulose content. HC, hemicellulose content. LC, lignin content. LRI, lodging resistance index. LR, lodging rate. P, direct path coefficient. IP, indirect path coefficient. P_A_, direct path coefficient of A on lodging resistance index. IP_AB_, indirect path coefficient of A on B. *, ** and *** denoted significance at *p* < 0.05 and *p* < 0.01 and *p* < 0.001 probability levels, respectively. (**A**) Correlation analysis; (**B**) path analysis.

Under the experimental conditions, the maize yield was significantly positively correlated with the grain number and 1000-grain weight. The diameter of the third internode, puncture strength, bending resistance, and the contents of cellulose, hemicellulose, and lignin, as well as the number of aerial root layers, were all positively correlated with grain yield (Figure 11A). Plant height, ear height, and center of gravity height were significantly negatively correlated with the lodging resistance index (LRI) and positively correlated with lodging rate. Maize yield was negatively correlated with the lodging rate.

Bending resistance (BR) had the largest direct effect on the LRI (0.776). The direct path coefficients of puncture strength (PS), cellulose (CC), hemicellulose (HC), and lignin (LC) were 0.266, 0.163, 0.004, and 0.193, respectively. Cellulose indirectly affected the LRI by influencing BR and PS, with indirect effect coefficients of 0.084 and 0.068, respectively. Lignin indirectly affected the LRI through BR and PS, with indirect effect coefficients of 0.141 and 0.173. Hemicellulose had no significant indirect effect on the LRI via BR and PS. Overall, BR contributed the most to lodging resistance, followed by PS (Figure 11B).

## 3. Discussion

### 3.1. Maize Yield Response to EDAH and Different Fertilization Combinations

**Figure 12:**
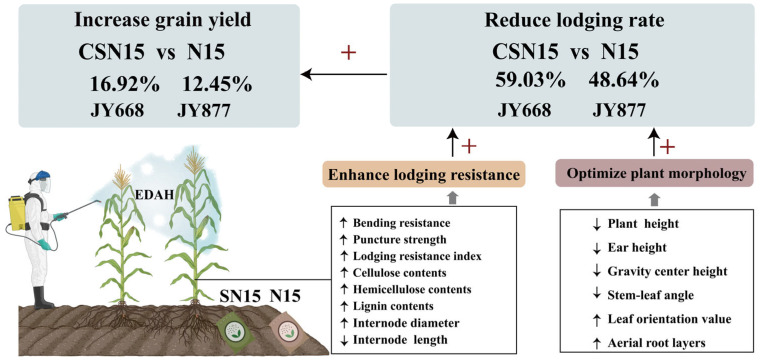
Response of lodging resistance and grain yield to EDAH and different fertilization combinations in spring maize.

Chemical regulation and fertilization management exerted significant impacts on maize yield [42,44]. Previous studies have explored the effects of chemical regulation on maize yield, and some research suggested that EDAH not only enhanced lodging resistance but also promoted grain filling [4,19] and increased the grain number and weight, thereby boosting yield [11,14,29]. This study demonstrated that spraying EDAH increased the grain number and weight, ultimately enhancing maize yield, which is consistent with previous findings. Other studies also showed that early application of EDAH in conditions with low lodging risk directs more assimilates to basal stems, resulting in thicker basal stems and enhanced lodging resistance; however, this process reduced dry matter allocation to ears, leading to fewer grains per ear and ultimately causing yield reduction [13,45]. Slow-release fertilizer was widely adopted to narrow the yield gap with potential maize yield and enhanced nutrient use efficiency, offering advantages in increasing grain production and reducing labor costs [46]. Slow-release fertilization synchronizes nitrogen release with crop demand and effectively reduces ammonia volatilization, nitrate leaching, and gaseous nitrogen losses, thereby improving fertilizer use efficiency and mitigating environmental risks associated with conventional nitrogen management [47,48]. The combined application of EDAH and slow-release nitrogen fertilization may improve nitrogen use efficiency, reduce fertilizer inputs, and decrease ammonia volatilization, nitrate leaching, and greenhouse gas emissions. A previous study using ^15^N labeling revealed that slow-release fertilizer aligned its nutrient release pattern with crop demand and significantly improved maize yield and fertilizer use efficiency compared to conventional fertilizer [49]. This study indicated that under the same N rate (225 kg/ha), one-time application of slow-release fertilizer improved maize lodging resistance and increased yield, consistent with previous studies. Furthermore, this experiment demonstrated that spraying EDAH did not significantly increase maize yield under the same fertilization treatment; however, the combined application of EDAH spraying and slow-release fertilization increased the grain number per ear and 1000-grain weight, ultimately improving maize yield compared with conventional fertilization (Figure 12).

### 3.2. Plant Morphology Response to EDAH and Different Fertilization Combinations

Spraying plant growth regulators improved plant morphological structure [11,50], decreased plant and ear height and gravity center height [18,29], and contributed to reducing the lodging rate of maize [51]. A possible reason was that lower ear height and gravity center height reduced the force exerted on the basal internode under external forces [52,53,54]. Upright leaf posture improved lodging resistance, while prostrated leaf posture reduced lodging resistance under high-density planting [27,55]. Upright leaf posture was conducive to forming a compact plant morphology, thereby effectively reducing the lodging rate [51]. Chemical regulation reduced stem–leaf angle above the ear leaf [51], thereby enhancing lodging resistance. EDAH increased the number, diameter, angle, volume, and dry weight of supporting roots, enhancing the root support capacity [4,14,33]. This study demonstrated that the combined application of EDAH and slow-release fertilization significantly reduced the stem–leaf angle, increased the leaf orientation value, and optimized leaf architecture, resulting in a more compact plant morphology and improved light distribution. In this study, EDAH combined with slow-release fertilization significantly promoted dry matter accumulation in the basal third internode (Table 1), primarily increased the diameter of the second to fourth internodes (Figure 6), and shortened the length of the second to fifth internodes (Figure 5), thereby reducing plant height and center of gravity, optimizing overall plant architecture, and significantly decreasing the lodging rate. The results of this experiment demonstrate that EDAH and slow-release fertilization significantly increased the number of aerial root layers and enhanced stem mechanical strength, which facilitated the efficient transport of water, nutrients, and photosynthetic products, further strengthened lodging resistance, and ultimately improved maize yield. Overall, the combined application of EDAH and slow-release fertilization optimized plant morphological structure and effectively improved maize lodging resistance, consistent with previous studies (Figure 12).

### 3.3. Lodging Resistance Response to EDAH and Different Fertilization Combinations

Lignin and cellulose contents, as major components of the cell wall, are crucial for plant vigor and resistance to biotic and abiotic stresses, including lodging [36]. The relative proportion of hemicellulose to cellulose influences the arrangement of microfibrils and the compactness of cell wall complexes, thereby modulating stem thickness, elasticity, and bending resistance [56,57,58,59]. Previous studies have shown that chemical regulation treatments can enhance the expression of enzymes involved in the biosynthetic pathways of lignin monomers, such as PAL, 4CL, and CAD, thereby increasing the contents of lignin, cellulose, and hemicellulose in the basal third internode of maize [60,61,62] and significantly reducing the lodging rate [4]. The results of this study indicate that the combination of spraying EDAH and slow-release fertilization significantly increased the cellulose, hemicellulose, and lignin contents in the basal internodes. Chemical components (cellulose, hemicellulose, and lignin) indirectly influence the lodging resistance index by affecting mechanical strength (bending resistance and puncture strength) (Figure 11). The mechanical strength of basal internodes increased after rapid elongation and thickening [21]. Applying EDAH during the optimal growth stage of the crop can modulate endogenous hormones and promote the expression of genes associated with secondary cell wall thickening [19,22], resulting in a shortened basal internode length [63], thereby increasing cell wall thickness and the degree of lignification and enhancing the mass of basal internode [17,51], which contributes to enhancing maize lodging resistance [12]. Slow-release fertilizer significantly increased the bending resistance and puncture strength of the second, third, and fourth internodes in maize [12], which also improved N supply–demand synchronization, providing a material basis for increased lignin, cellulose, and hemicellulose contents [22,64,65]. This effect not only results from a stable nitrogen supply but is also closely associated with the nitrogen-mediated coupling of carbon and nitrogen metabolism in the phenylpropanoid pathway during lignin biosynthesis [59]. An adequate and balanced nitrogen supply promotes the synthesis of amino acids (such as phenylalanine), thereby providing the basis for the accumulation of lignin precursors [62], and increased the mass density of the internodes [5,58]. Our study indicated that EDAH combined with slow-release fertilization increased the dry weight per unit length of the basal third internode, leading to stem thickening and further enhancing the puncture strength and bending resistance of the third internode. This consequently reduced the lodging rate and increased the lodging resistance index, with bending resistance having the largest direct effect on the lodging resistance index (Figure 11). Furthermore, this treatment significantly increased the contents of cellulose, hemicellulose, and lignin in the basal internodes. Changes in these structural carbohydrates were significantly positively correlated with bending resistance and puncture strength, with lignin exerting the greatest indirect effect on the lodging resistance index through bending resistance and puncture strength (Figure 11), indicating their key role in improving stem mechanical strength. Overall, the combined application of EDAH and slow-release fertilization enhanced stem chemical composition and mechanical strength, ultimately improving maize lodging resistance (Figure 12).

## 4. Materials and Methods

### 4.1. Experimental Site

The field experiments were conducted at Yangzhou University experimental farm (Yangzhou, Jiangsu Province) from 2023 to 2025. Experimental soil was sandy loam, and the pH, organic matter, total N, alkali-hydrolyzable N, available phosphorus, and available potassium in tillage layer (0–20 cm) were 7.5, 12.0 g/kg, 0.7 g/kg, 67.3 mg/kg, 24.8 mg/kg, and 43.1 mg/kg in 2023; 7.1, 12.3 g/kg, 0.8 g/kg, 70.5 mg/kg, 24.3 mg/kg, and 40.9 mg/kg in 2024; and 7.6, 12.5 g/kg, 0.9 g/kg, 80.5 mg/kg, 26.3 mg/kg, and 43.9 mg/kg in 2025. Meteorological data during the maize growing periods are shown in Figure 2. The total accumulated temperature and precipitation during the growing season were 2478.8 °C and 678.5 mm in 2023, 2467.0 °C and 499.8 mm in 2024, and 2715.6 °C and 383.6 mm in 2025. Temperature and precipitation data were obtained from the NASA POWER Data Access Viewer (DAV) (https://power.larc.nasa.gov) for the period of 2023–2025. Daily maximum, minimum, and average temperatures, as well as daily precipitation, were used to evaluate the effects of environmental conditions on maize growth, yield, and lodging resistance under different treatments.

### 4.2. Experimental Design

Two maize varieties, Jiangyu668 (JY668) and Jiangyu877 (JY877), were selected as experimental materials. Plant heights in the approval announcement of JY668 and JY877 were 223 and 246 cm, and ear heights were 96.1 and 96.0 cm. Treatments were arranged in a split–split plot design in the three-year experiment. Two maize varieties represented the main plot, and the split plot was assigned with spraying EDAH and no EDAH. Fertilization treatments, comprising no fertilization (N0), conventional fertilization (N15), and slow-release compound fertilization (SN15), were assigned to the split–split plot level of the experimental design. EDAH (27% ethephon and 3% DA-6), sourced from Anhui Lantian Agricultural Development Co., Ltd., Hefei, China, was uniformly applied to all maize leaves at the eighth extended leaf stage with a concentration of 3.75 mL/L. N15 treatment was applied at a concentration of 500 kg/ha conventional compound fertilizer (N-P_2_O_5_-K_2_O = 15-15-15) at sowing and 326 kg/ha conventional urea (N = 46%) at the sixth extended leaf stage. SN15 was applied at a concentration of 833.5 kg/ha slow-release compound fertilizer (N-P_2_O_5_-K_2_O = 27-9-9) at the sowing stage. All fertilizers were supplied by Jiangsu Zhongdong Fertilizer Co., Ltd. (Changzhou, Jiangsu, China). Each plot was 10 m long and 9.6 m wide with 16 rows (spacing of 0.4 and 0.8 m). The planting density was 75,000 plants/ha. Seeds were sown at a depth of 3–5 cm on 13 April 2023, 10 April 2024, and 8 April 2025. The harvest dates were 25 July 2023, 23 July 2024, and 26 July 2025, respectively. The depth of furrow fertilization was 10 cm. Other field management followed farmers’ conventional practices. Weed control was implemented using a combination of a pre-emergence residual herbicide and a single post-emergence application at the fifth leaf stage. All maize seeds were treated with Acceleron™ (containing difenoconazole, fludioxonil, metalaxyl-M, and thiamethoxam) prior to sowing, and phoxim was applied to the soil of each plot at sowing to control corn rootworms [46].

### 4.3. Sampling and Measurements

#### 4.3.1. Grain Yield and Its Components

At maturity, three sampling points were selected in each plot, and 10 consecutive ears were harvested from each point. A total of 30 harvested ears were air-dried and hand-threshed. Measurements included 1000-grain weight, grain number per ear, and moisture content. Grain yield was calculated and adjusted to a standard moisture content of 14%.

#### 4.3.2. Plant Morphology

Ten consecutive plants were selected from each plot to measure the plant height, ear height, center of gravity height, length, and diameter of the third basal internode at silking stage (R1). Then, ten plants were cut at the base and tied at their center using a thin string. The string position was adjusted until the plant remained perfectly horizontal, with the distance from the base to the string’s position recorded as the center of gravity height. As maize stems are elliptical, internode diameter was determined using a vernier caliper by recording both the major and minor axes of internode cross-section, and the average of these two values represented the third internode diameter. At the milk stage (R3), 30 consecutive plants were selected from each plot to determine the number of aerial root layers, and 10 consecutive plants were selected to determine the leaf–stem angle and leaf orientation value. Leaf–stem angles (*A*) were measured using a protractor at ear leaf, above ear leaf, and below ear leaf. *A* measuring tape was used to record the straight-line distance from the leaf base to the highest point of the leaf (*Lf*) and the leaf length (*L*). These measurements were used to calculate the leaf orientation value.

(1) $$Leaf\;orientation\;value\left(LOV\right)=\left(90{}^{\circ}-A\right)\times \frac{Lf}{L}$$

#### 4.3.3. Stem Mechanical Properties and Lodging Rate

Three representative and uniformly grown plants per plot were selected to measure puncture strength by a portable plant lodging resistance tester (Zhejiang Top Cloud-agri Technology Co., Ltd., Hangzhou, China), which was specifically designed for assessing crop lodging resistance. The puncture probe measured stem puncture strength at upper, middle, and lower sections of the third internode, aligned perpendicular to the stem. The highest value recorded during probe penetration of stem epidermis was defined as puncture strength. After leaves, leaf sheaths, stems, and ears were removed, the bending resistance was measured immediately. During measurement, prepared stems were positioned on a stand designed for a lodging resistance tester. A bending resistance probe applied a uniform and gradual downward force at the midpoint of the third internode from base [42]. The maximum value recorded at point of internode failure was defined as bending resistance. The lodging resistance index was calculated based on bending resistance and the height of the stem gravity center [12]. After testing, third internodes from the base were individually collected, oven-dried at 60 °C, and ground into a powder for analyzing cellulose, hemicellulose, and lignin contents according to a previous study [17]. At maturity, three central double rows were selected for investigation. The number of plants exhibiting stem lodging and root lodging was recorded separately, and the lodging rate was calculated [11].

(2) Lodging rate(%) = (Number of lodged plants/Total number of plants per plot) × 100%

(3) Lodging resistance index=bendingresistance of basal internodes / center of gravity height

### 4.4. Statistical Analysis

Statistical analysis and significance tests were performed using SPSS 27 (IBM Corp., Armonk, NY, USA). Multiple comparisons among treatment means were performed using Tukey’s HSD post hoc test. Data organization was conducted using Excel 2019 (Microsoft Corp., Redmond, WA, USA). Correlation analysis between lodging resistance-related traits and yield was carried out using Origin 2021 (Origin Lab Corp., Northampton, MA, USA), which was also utilized to create visualization graphs for experimental indicators.

## 5. Conclusions

This study demonstrated that the combined application of EDAH spraying and slow-release fertilization improved maize plant morphology, enhanced lodging resistance, and increased grain yield (Figure 12). This integrated strategy lowered the plant gravity center and stem–leaf angle, optimized leaf spatial distribution, and increased internode diameter and the number of aerial root layers. It also promoted the accumulation of structural components, including cellulose, hemicellulose, and lignin, thereby strengthening stem mechanical properties and increasing the lodging resistance index. These improvements reduced lodging occurrence and enhanced the grain number and weight. Overall, this combined management approach effectively improved lodging resistance and yield potential in maize, providing theoretical and technical support for green and high-yield maize production. This integrated management strategy may also have potential applicability in other maize-growing regions, although its broader effectiveness requires further verification under diverse environmental conditions.

## Figures and Tables

**Table 1:** Response of maize yield and its components to EDAH and different fertilization combinations in maize from 2023 to 2025.

Year	Variety	Treatment	Row Number	Grains Per Row	Grain Number Per Ear	1000-Grain Weight (g)
2023	JY668	N0	14.67c	28.25b	414.33c	252.46c
		N15	15.33ab	30.58ab	468.94bc	267.95b
		SN15	14.67c	33.83a	496.17b	284.56a
		CN0	14.67c	27.42b	402.11c	255.56c
		CN15	15.33ab	31.25ab	479.17bc	270.28ab
		CSN15	16.67a	30.94ab	515.11a	278.38ab
	JY877	N0	15.33ab	27.69b	424.58c	264.13b
		N15	16.44a	30.86ab	507.67ab	274.66ab
		SN15	16.22a	31.86ab	516.84a	274.51ab
		CN0	15.56ab	26.53b	412.94c	266.14b
		CN15	16.00a	31.89ab	510.24a	276.24ab
		CSN15	15.33ab	33.75a	517.33a	286.37a
2024	JY668	N0	13.78b	29.33b	404.15bc	262.28c
		N15	14.22a	32.04a	455.64ab	273.79b
		SN15	14.44a	31.37ab	453.13ab	300.66a
		CN0	13.56b	29.15b	395.17c	262.73c
		CN15	13.44b	34.04a	457.63ab	280.60ab
		CSN15	14.22a	33.70a	479.34a	289.19ab
	JY877	N0	13.67b	29.85b	407.98bc	264.02c
		N15	13.22b	32.93a	435.35b	289.82ab
		SN15	14.00a	33.33a	466.67ab	284.34ab
		CN0	14.00a	28.63b	400.81bc	264.18c
		CN15	14.00a	33.15a	464.07ab	280.13ab
		CSN15	14.22a	33.82a	480.92a	294.73a
2025	JY668	N0	15.40c	35.10b	540.20d	231.10b
		N15	15.00c	39.33a	587.33c	239.08b
		SN15	15.40c	39.87a	610.33c	266.90a
		CN0	16.40b	28.57d	467.93e	231.95b
		CN15	15.40c	38.77b	596.00c	227.76b
		CSN15	16.20b	40.53a	656.40b	266.73a
	JY877	N0	16.60b	33.43c	555.00d	218.11c
		N15	16.00b	36.97b	607.93c	253.28ab
		SN15	17.00a	39.27a	667.67a	271.60a
		CN0	16.60b	32.80c	550.74d	208.52c
		CN15	16.80b	36.33b	611.07c	236.05b
		CSN15	18.20a	37.07b	674.00a	254.70ab
ANOVA						
Years (Y)		2023	15.52b	30.40c	472.12b	270.94b
		2024	13.90c	31.78b	441.74c	278.87a
		2025	16.25a	36.50a	593.72a	247.15c
Variety (V)		JY668	14.93b	33.00a	493.28b	263.44b
		JY877	15.51a	32.79b	511.77a	264.53a
Fertilization (F)		N0	15.02b	29.73c	447.99c	248.43c
		N15	15.10a	34.01a	515.09b	264.14b
		SN15	15.55a	34.94a	544.49a	279.39a
Chemical regulation (C)		C0	15.08b	33.10a	501.11b	265.18a
		C	15.37a	32.68b	503.94a	262.79b
Years (Y)			145.96 **	350.63 **	349.42 **	397.35 **
Varieties (V)			27.27 **	0.94	12.69 **	5.25 *
Chemical regulation (C)			7.54 **	4.33 *	0.72	2.76
Fertilization (F)			7.45 **	247.01 **	122.57 **	425.74 **
Y × V			12.23 **	3.97 *	3.73 *	3.83 *
Y × C			4.18 *	10.58 **	0.24	1.33
Y × F			2.29	6.17 **	5.52 **	23.28 **
V × C			0.14	0.79	0.05	0.32
V × F			0.02	1.01	0.16	13.54 **
C × F			1.57	10.96 **	4.48 *	2.46
Y × V × C			5.66 **	5.73 **	0.30	3.35 *
Y × V × F			2.27	5.89 **	0.97	15.46 **
Y × C × F			0.49	1.17	1.40	3.96 **
V × C × F			2.30	2.53	2.77	9.08 **
Y × V × C × F			2.36	8.12 **	1.23	2.99

Note: Values are presented as mean ± SD (n = 9). N0: no fertilization; N15: conventional fertilization; SN15: one-time application of slow-release fertilizer; CN0: spraying EDAH under no fertilization; CN15: spraying EDAH under conventional fertilization; CSN15: spraying EDAH under one-time application of slow-release fertilizer; C: Chemical regulation; C0: no chemical regulation. Multiple comparisons among treatment means were performed using Tukey’s HSD post hoc test. Different letters indicate significant differences among treatments. * and ** denote significance at *p* < 0.05 and *p* < 0.01 probability levels, respectively.

**Table 2:** Response of maize third internode traits to EDAH and fertilization from 2023 to 2025.

ANOVA		2023				2024				2025				
		TDW (g)	TIL (cm)	TSD (mm)	LDW (g/cm)	TDW (g)	TIL (cm)	TSD (mm)	LDW (g/cm)	TDW (g)	TIL (cm)	TSD (mm)	LDW (g/cm)	
Variety (V)	JY668	5.76a	12.55b	24.27b	0.46a	4.11a	9.16a	22.20b	0.45a	4.11a	9.16a	22.20b	0.45a	
	JY877	5.50b	12.65a	24.42a	0.43b	3.76b	8.47b	25.81a	0.44b	7.12a	9.6a	28.06a	0.78a	
Fertilization (F)	N0	4.47c	12.02c	23.55c	0.37c	2.91c	7.95c	20.18c	0.37b	4.78c	6.83b	21.95c	0.70b	
	N15	5.68b	12.55b	24.19b	0.46b	4.13b	8.77b	25.08b	0.48a	5.55b	6.78c	26.74b	0.82a	
	SN15	6.73a	13.23a	25.30a	0.51a	4.77a	9.73a	26.75a	0.49b	6.77a	10.72a	28.73a	0.64b	
Chemical regulation (C)	C0	4.98b	12.88a	23.87b	0.39b	3.63b	9.26a	23.41b	0.39c	5.28b	8.67a	24.56b	0.59b	
	C	6.27a	12.32b	24.82a	0.51a	4.24a	8.37b	24.6a	0.50a	6.12a	7.55b	27.05a	0.85a	
F-value														
Varieties (V)		1.71	0.32	1.01	1.46	0.85	5.32 *	47.45 **	0.01	131.88 **	203.76 **	346.506 **	0.00	
Chemical regulation (C)		41.74 **	10.23 **	43.68 **	41.67 **	2.67	8.86 **	5.13 *	5.58 *	11.51 **	28.94 **	106.598 **	95.09 **	
Fertilization (F)		42.98 **	15.80 **	50.52 **	18.47 **	8.46 **	11.92 **	56.60 **	3.03	21.99 **	156.02 **	276.267 **	3.35	
V × C		1.42	0.06	7.87 *	0.95	0.00	0.04	0.08	0.01	7.51 *	3.48	7.343 *	0.00	
V × F		1.47	0.43	10.76 **	0.80	2.54	3.34	12.90 **	0.77	5.42 *	1.82	5.319 *	0.00	
C × F		5.43 *	0.86	14.7 **	3.32	0.02	0.19	1.29	0.03	4.47 *	0.19	3.732 *	14.02 **	
V × C × F		0.82	0.44	11.54 **	0.52	0.24	0.75	0.42	0.04	0.87	0.87	10.889 **	0.00	

Note: TDW: third internode dry weight; IL: length of third internode; SD: diameter of third internode stem; LDW: dry weight per unit length; LDW = TDW/IL [4]; N0: no fertilization; N15: conventional fertilization; SN15: one-time application of slow-release fertilizer; C: chemical regulation; C0: no chemical regulation. Multiple comparisons among treatment means were performed using Tukey’s HSD post hoc test. Lowercase letters indicate the significance at *p* < 0.05. * and ** denote significance at *p* < 0.05 and *p* < 0.01 probability levels, respectively.

## Data Availability

Informed consent was obtained from all subjects involved in the study. All the data and code used in this study can be requested by email to the corresponding author, Guanghao Li, at guanghaoli@yzu.edu.cn.
